# Autonomous Droplet Microfluidic Design Framework with Large Language Models

**DOI:** 10.1021/acsomega.5c06253

**Published:** 2025-09-26

**Authors:** Dinh-Nguyen Nguyen, Raymond Kai-YuTong, Ngoc-Duy Dinh

**Affiliations:** Department of Biomedical Engineering, 26451 The Chinese University of Hong Kong, Shatin, New Territories 999077, Hong Kong

## Abstract

Droplet-based microfluidic devices have substantial promise as cost-effective alternatives to current assessment tools in biological research. Moreover, machine learning models that leverage tabular data, including input design parameters and their corresponding efficiency outputs, are increasingly utilized to automate the design process of these devices and to predict their performance. However, these models fail to fully leverage the data presented in the tables, neglecting crucial contextual information, including column headings and their associated descriptions. This study presents μ-Fluidic-LLMs, a framework designed for processing and feature extraction, which effectively captures contextual information from tabular data formats. μ-Fluidic-LLMs overcomes processing challenges by transforming the content into a linguistic format and leveraging pretrained large language models (LLMs) for analysis. We evaluate our μ-Fluidic-LLMs framework on prediction tasks utilizing publicly available data sets on droplet microfluidics. We demonstrate that our μ-Fluidic-LLMs framework can empower deep neural network models to be highly effective and straightforward while minimizing the need for extensive data preprocessing. When combined with LLMs like LLAMA3.1 and DEEPSEEK-R1, deep neural networks achieve marked improvements, lowering the mean absolute error in generation rate by nearly 40%, reducing the root mean squared error in droplet diameter by around 26%, and enhancing regime classification accuracy by over 3% in comparison with prior results. This study lays the foundation for the huge potential applications of LLMs and machine learning in a wider spectrum of microfluidic applications.

## Graphical abstract

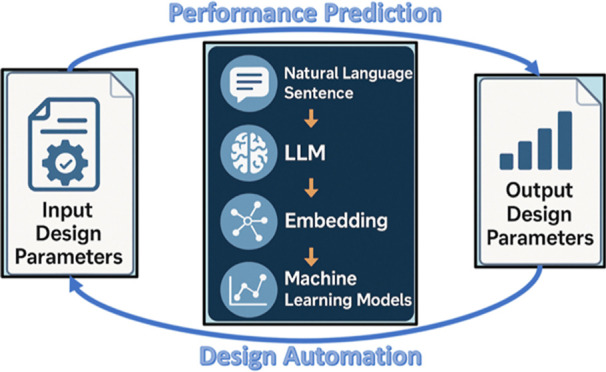

## Introduction

Droplet-based microfluidics has been recognized as a groundbreaking technology for miniaturising biological and chemical experiments. It has significantly advanced biotechnology^1–8^ by enabling techniques such as next-generation sequencing,^9–11^ single-cell RNA sequencing,^11–13^ droplet digital PCR,^14–17^ and liquid biopsies diagnostics.^18^ However, the impact of microfluidics remains largely confined to single-use cartridges, integrated benchtop devices, and specialized lab setups.^19,20^ In addition, the complex design and fabrication of custom microfluidic devices have limited their widespread adoption and general use.^21,22^ Moreover, microfluidic design and operation can take months or years of iterative testing to optimize, even if fabrication is outsourced at a high cost.^23^ To overcome these limitations, machine learning, which predicts patterns and behavior, has been employed. Machine learning models are becoming more popular for predicting performance and automating design in microfluidic droplet generation. For instance, Mahdi and Daoud^24^ used machine learning to predict the size of water droplets in glycerine oil from a T-junction setup. The model, trained with 742 data points, accurately predicted droplet size using Reynolds and capillary numbers across different flow rates and fluid properties within one geometry. Furthermore, in Lashkaripour et al.,^25^ neural networks were used to predict droplet size, generation rate, and flow regime based on design geometry and flow conditions. The neural networks, which were trained on 888 data points with varying capillary numbers, flow rate ratio, and six geometric parameters, accurately predicted the droplet generation regime (95.1% accuracy), size (error <10 μm), and generation rate (error <20 Hz) for droplets ranging from 25 to 250 μm in size and 5 to 500 Hz in rate. Elsewhere, in Damiati et al.,^26^ a machine learning model predicted the size of poly­(lactic-*co*-glycolic acid) (PLGA) microparticles produced by flow-focusing droplet generators and dichloromethane solvent evaporation. The model was trained on data from 223 combinations of flow rates, PLGA concentrations, device types, and sizes to predict PLGA particle size (*R* ^2^ > 0.94) accurately. Furthermore, Hong et al.^27^ applied machine learning models to automate the design of concentration gradient generators. A neural network trained on 9 million data points from a verified physics model was able to map desired concentration profiles to inlet settings, achieving an 8.5% error rate. Meanwhile, Ji et al.^28^ applied machine learning to automate the iterative design of grid micromixers. Neural networks were trained on 4320 simulated chips to map channel lengths to output concentrations. The designs met outlet concentration targets within 0.01 mol/m^3^, compared with simulations, for 91.5% of benchmarks. Moreover, Dressler et al.^29^ compared two reinforcement learning algorithms, Deep-Q Networks (DQNs) and model-free episodic controllers (MFECs), in controlling laminar flow between fluids and droplet generation in water-in-oil emulsions. Both models achieved or exceeded superhuman performance and can be adapted to optimize complex systems, such as double emulsions or liposome formation. However, these machine learning models are limited to processing the explicit content within tables, without considering the surrounding contextual information, such as column headers and accompanying descriptions. Furthermore, the data processing becomes more complex when inconsistencies in units of measurement or data types are present across varying tabular data systems. These challenges could be mitigated by harnessing the power of language-based approaches, because language is a highly versatile data modality capable of representing information across diverse data points without the need for structural consistency. Additionally, recent advancements in large language models (LLMs) utilizing Transformer architecture^30^ have enabled state-of-the-art performance across a diverse array of language tasks, including translation, sentence completion, and question answering. These pretrained models are frequently built using vast and diverse data sets, which allows them to leverage prior knowledge and make accurate predictions even with minimal training data. Some LLMs are specifically trained to address domain-specific knowledge and technical complexities, enhancing their utility in relevant applications. For example, Boiko et al.^31^ introduced a LLM–driven agent that autonomously designs, plans and executes experiments by using web search, documentation and robot APIs. In addition, Bran et al.^32^ used GPT-4 augmented with 18 chemistry tools to plan and execute syntheses on a cloud-connected robotic platform. Furthermore, Yoshikawa et al.^33^ proposed **CLAIRIFY**, which combined LLMs with task-and-motion planning to translate natural-language instructions into safe robot actions in a chemistry lab. Moreover, Park et al.^34^ created a corpus of 1.5 million AI-generated *materials narratives*, enabling LLMs to propose new compounds through inverse design. Additionally, Ghafarollahi and Buehler^35^ developed a multiagent LLM system to **design** denovo **proteins**, integrating specialized agents for structure analysis and molecular simulations. Jia et al.^36^ also demonstrated an AI-enhanced active-matrix digital microfluidic (AM-DMF) platform for single-cell sample manipulation, in which an LLM generates droplet-routing plans while a vision system tracks droplets, enabling fully automated cell-handling workflows. Moreover, LLMs are being explored for automating diagnostic and therapeutic workflows,^37^ medical investigations,^38–40^ and scientific discovery.^41^

In this study, we establish μ*-Fluidic-LLMs*, a systematic framework that utilizes linguistic techniques to derive contextual information related to droplet microfluidic design parameters within tabular structures. This approach produces more comprehensive data representations. We handle tabular data by converting each data sample into a corresponding textual representation. This representation is integrated into the column attributes with their respective values while incorporating any relevant contextual information that may be present. Subsequently, the complete text is fed into a pretrained LLM, which generates fixed-dimensional embeddings from its final layer. These embeddings are then utilized as input for standard machine learning models to perform downstream tasks. Then, we evaluate the effect on final task performance by comparing the performance when inputting the embeddings into standard machine learning models against the performance when inputting the original tabular features into the same models. Finally, we demonstrate that integrating deep neural networks (DNNs) with LLMs substantially enhances performance on downstream tasks. We also demonstrate the adaptability of this framework, enabling it to incorporate new LLMs as they become available. An overview of this study is illustrated in Figure 1.

**Figure 1:**
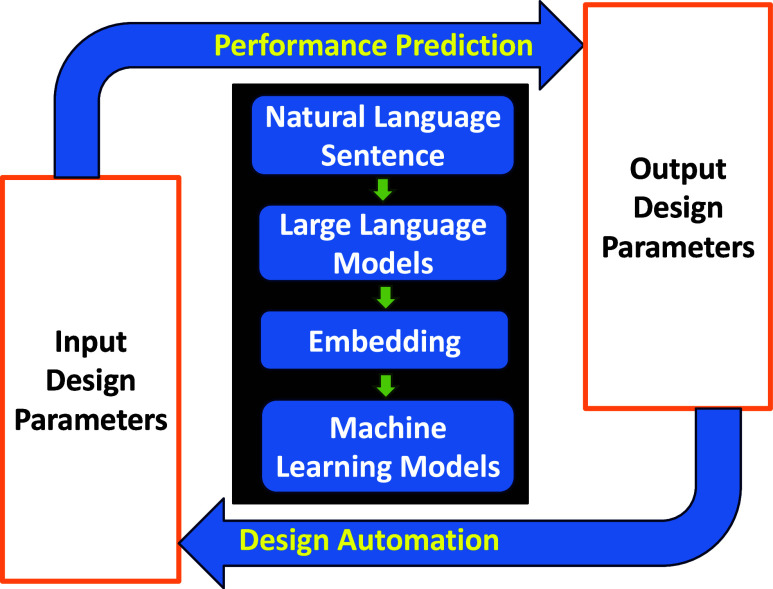
μ-Fluidic-LLMs framework. Regarding performance prediction, μ-Fluidic-LLMs transforms input design parameters from tabular structures into natural language sentences, which are then processed by LLMs to generate embeddings from the final layer. These embeddings are subsequently used as input for traditional machine learning models. In the context of design automation, μ-Fluidic-LLMs follows the same steps; however, the output design parameters serve as the input parameters.

## Experimental Section

The overall method is illustrated in Figure 2, including two modules such as performance prediction (Figure 2A) and design automation (Figure 2B). Figure 2A illustrates the process of converting each row of tabular data into a paragraph that includes the eight column headers and their corresponding values. This paragraph is then input into large language models to generate embeddings from the final layer, which are subsequently used in machine learning models for performance prediction. Figure 2B illustrates a similar procedure for design automation, with the paragraph containing only three column headers and their corresponding values.

**Figure 2:**
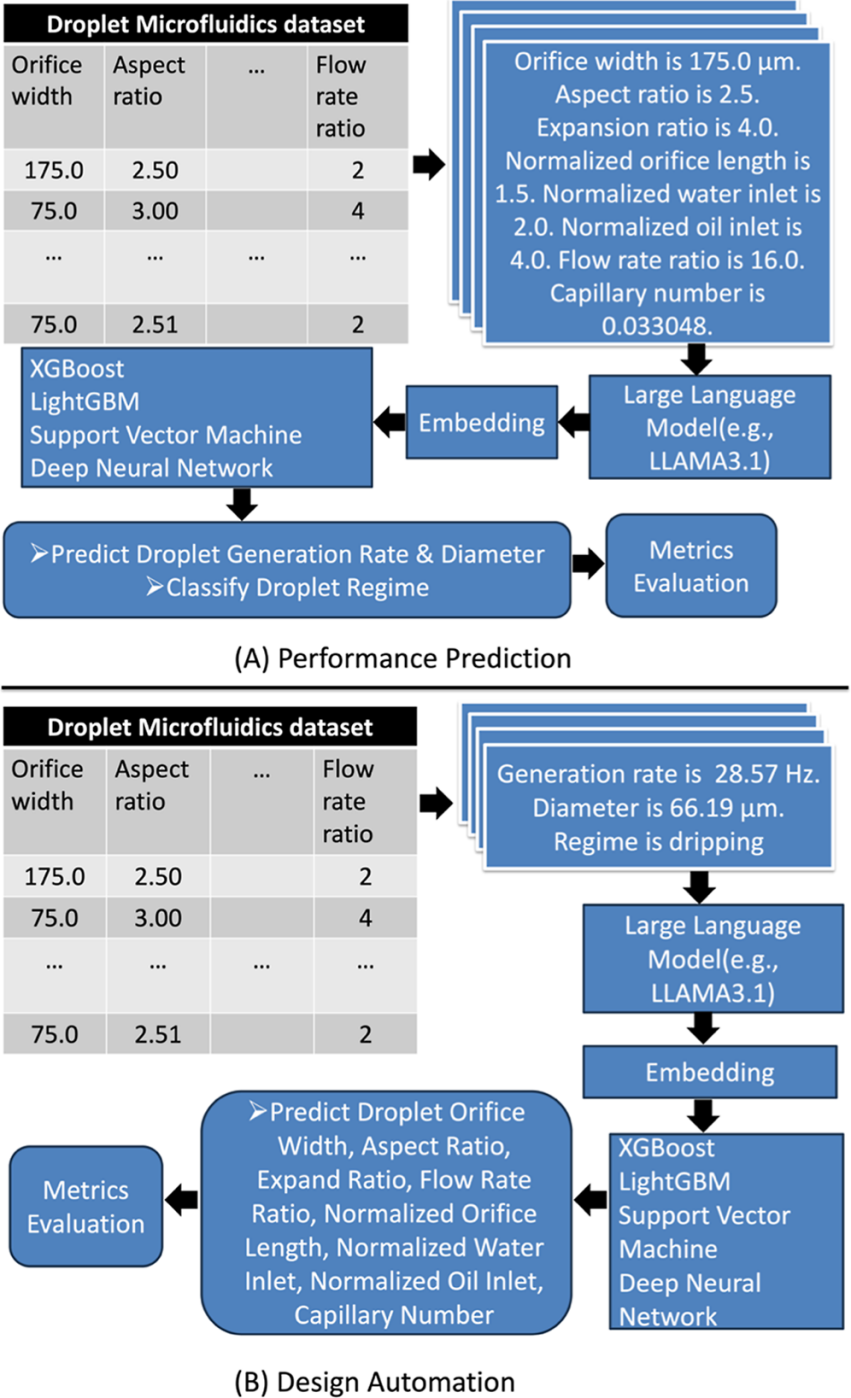
Summary of the overall methodology. Figure 2A illustrates the process of converting each row of tabular data into a paragraph that includes the eight column headers and their corresponding values. Figure 2B demonstrates a similar procedure for design automation.

### Data and Tasks

To evaluate model performance, we employed two data sets related to droplet microfluidics. The first data set,^25^ comprising 998 tabulated entries, pertains to flow-focusing droplet generation and includes 11 features: orifice width, normalized orifice length, normalized water and oil inlet widths, expansion ratio, aspect ratio, flow rate ratio, capillary number, droplet diameter, generation rate, and flow regime. Using this data set, we performed two regression tasks and one classification task aimed at predicting system performance, and the results were benchmarked against prior studies. Additionally, a regression task was conducted to demonstrate the design automation capability.

The second data set,^42^ consisting of 868 entries, also relates to droplet microfluidics and includes 9 features: orifice width, normalized channel depth, flow rate ratio, capillary number, normalized continuous and dispersed inlets, normalized outlet width, observed generation rate, and observed droplet diameter. Two regression tasks were conducted using this data set to assess predictive performance, and the outcomes were compared with previous work.

### Text Serialisation for Performance Prediction and Design Automation

Text serialisation is the process of converting structured data into a linear, text-based format that can be easily stored, transmitted, and reconstructed. In LLMs, text serialisation plays a vital role in processing and managing vast amounts of data.^43^ These models rely on serialised text data to train on diverse data sets, often including a mix of natural language, structured information, and metadata. By converting complex input data into a serialised text format, LLMs can uniformly process this information, enabling them to generate meaningful predictions, responses, or completions based on the input. Furthermore, the serialised text is essential for fine-tuning models with specific data, allowing the LLMs to adapt to new tasks or domains by processing serialised input and output pairs.^44,45^ Our framework implements manual text serialisation for individual data samples (Figure 2). By leveraging the column headers of tabular data, the framework systematically transforms each table row into a sequence of key–value pairs, with the keys originating from the column headers and the values corresponding to the respective data entries. For performance prediction (Figure 2A), the eight column headers and their corresponding values are transformed into a paragraph. This text is then input into pretrained LLMs to generate embeddings from the final layer. These embeddings are subsequently used as input for baseline machine learning models, which predict output values such as droplet diameter, generation rate, and regime. For design automation (Figure 2B), the three column headers droplet diameter, generation rate, and regime, along with their respective values, are transformed into a paragraph. This paragraph is processed by pretrained LLMs to produce embeddings from the final layer, which are then utilized as inputs for the baseline machine learning models to predict the remaining eight design parameters. This strategy is designed to maintain both the integrity and the contextual meaning of the original tabular data during serialisation.

Feature values in the data set are generally expressed in the format “*X* is *V* [unit],” where *X* denotes the feature name (e.g., orifice width or flow rate ratio), *V* represents the numerical value assigned to that feature, and unit corresponds to the physical measurement unit, such as micrometres (μm), hertz (Hz), or dimensionless ratios, depending on the feature type. This standardized phrasing facilitates consistent interpretation and machine-readability across different data records. In instances where the value of a feature is not available due to missing measurements, experimental limitations, or irrelevance to a particular data entry, this absence is explicitly communicated using the phrase “*X* is missing.” Alternatively, if appropriate for the application, the missing feature may be omitted entirely from the textual representation. When a feature value is defined not by a single measurement but rather by a range of possible values, this information can be conveyed in one of two standard formats. The first, “*X* belongs to a range of *Y* [unit] and *Z* [unit],” indicates that the feature value lies somewhere within the interval bounded by *Y* (the lower limit) and *Z* (the upper limit). The second, more descriptive variant, “*X* is greater than *Y* [unit] and smaller than *Z* [unit],” may be preferred when emphasizing constraint boundaries or when precise inclusion/exclusion criteria must be maintained. These representations enable more nuanced encoding of uncertainty or variability in experimental parameters. Collectively, these conventions ensure that both quantitative completeness and semantic clarity are maintained throughout the data set, thereby supporting downstream analyses, reproducibility, and interpretability in both human- and machine-readable contexts.

### Text Embeddings

Text embedding is a technique used to convert textual data into a dense, fixed-size vector representation. These vectors capture the semantic meaning of the text by mapping words, phrases, or entire documents into a continuous vector space, where similar pieces of text are represented by vectors that are close to each other. The primary advantage of text embedding lies in its ability to represent complex linguistic relationships and contextual meanings in a numerical format that can be efficiently processed by machine learning algorithms. This makes text embedding an essential component in various natural language processing (NLP) tasks, such as sentiment analysis, text classification, and machine translation.^46^

### Large Language Model Selection

LLMs play a pivotal role in generating high-quality text embeddings by leveraging their understanding of linguistic patterns and contextual relationships. Pretrained LLMs are particularly effective in producing embeddings because they are trained on vast text corpora and can capture nuanced semantic information. These models transform input text into embeddings by processing it through multiple layers of neural networks, where each layer refines the representation by focusing on different aspects of the language, such as syntax, semantics, and context. The resulting embeddings are highly informative and can be used as input for downstream NLP tasks, enhancing the performance of models in applications like question-answering document retrieval and text summarization.

We employed five widely used open-source foundational LLMs, which provide full access to their architectures and weights, thereby supporting transparency and reproducibility.

This open-access model eliminates dependence on costly commercial APIs, offering a more scalable and financially viable solution for sustained experimentation. Our approach embodies a broader commitment to methodological openness, economic sustainability, and long-term reproducibility. A detailed list of the LLMs used is provided in Table 1.

**Table 1:** LLMs Utilized

model	model size	vendor	license	source
DEEPSEEK-R1	8B	DeepSeek	open	^47^
LLAMA3.1	8B	Meta	open	^48^
MISTRAL	7B	Mistral AI	open	^49^
GEMMA2	9B	Google	open	^50^
LLAVA	7B	Microsoft	open	^51^

### Baseline Machine Learning Models

To assess the effectiveness of text embeddings compared with nontext embeddings in downstream tasks, we employed a range of standard machine learning models, including XGBoost,^52^ LightGBM,^53^ and support vector machine (SVM).^54^ Additionally, DNNs, an advanced form of traditional neural networks characterized by the addition of multiple hidden layers,^55^ were designed and applied in this study. We subsequently optimized the hyperparameters for all baseline models and their corresponding combinations with pretrained large language models. The data set was partitioned into 80% for training, 10% for validation, and 10% for testing.

### Evaluation Metrics

After finalising the models, we performed a repeated 10-fold cross-validation 15 times to obtain more robust and reliable mean performance estimates, thereby reducing the variability introduced by random data partitioning.^56^ For regression tasks, the evaluation utilized four metrics: mean absolute error (MAE), mean squared error (MSE), root mean squared error (RMSE), and the coefficient of determination (*R* ^2^), along with their associated standard errors. For the classification task, five metrics were employed: accuracy, F1 score, precision, recall, and area under the receiver operating characteristic curve (ROC AUC), each accompanied by standard errors. Here, we present line graphs comparing MAE across models for regression and accuracy across models for classification. Line graphs for the remaining metrics are included in the Supplementary File. In addition, we provide tables in the “Results & discussion” section demonstrating the minimization of standard error and the stabilization of mean estimated performance for metrics across different models. Specifically, the values presented in the table are those obtained from repetitions in which the discrepancy between the mean and median of the corresponding metric is minimized, indicating that the data likely follows a normal distribution.^56,57^

## Results and Discussion

### Performance in Prediction of Droplet Diameter

To assess the trends in accuracy in droplet diameter prediction of first data set^25^ across different models, a detailed comparison of 24 models over 15 repetitions was conducted, using MAE as the performance metric, as illustrated in Figure 3. Among all configurations, the DNN-LLAMA3.1 model consistently achieved the lowest MAE, around 8.56 across repetitions, followed closely by DNN-LLAVA. Notably, baseline models trained without pretrained embeddings showed moderate performance further underscoring the substantial benefit conferred by semantically enriched embeddings. These results highlight the critical role of embedding selection in regression tasks, suggesting that LLAMA3.1 offers a more information-dense representation of input features, thereby enhancing model generalization and accuracy. Such findings reinforce the growing utility of LLMs in downstream scientific applications requiring high-precision numerical prediction.

**Figure 3:**
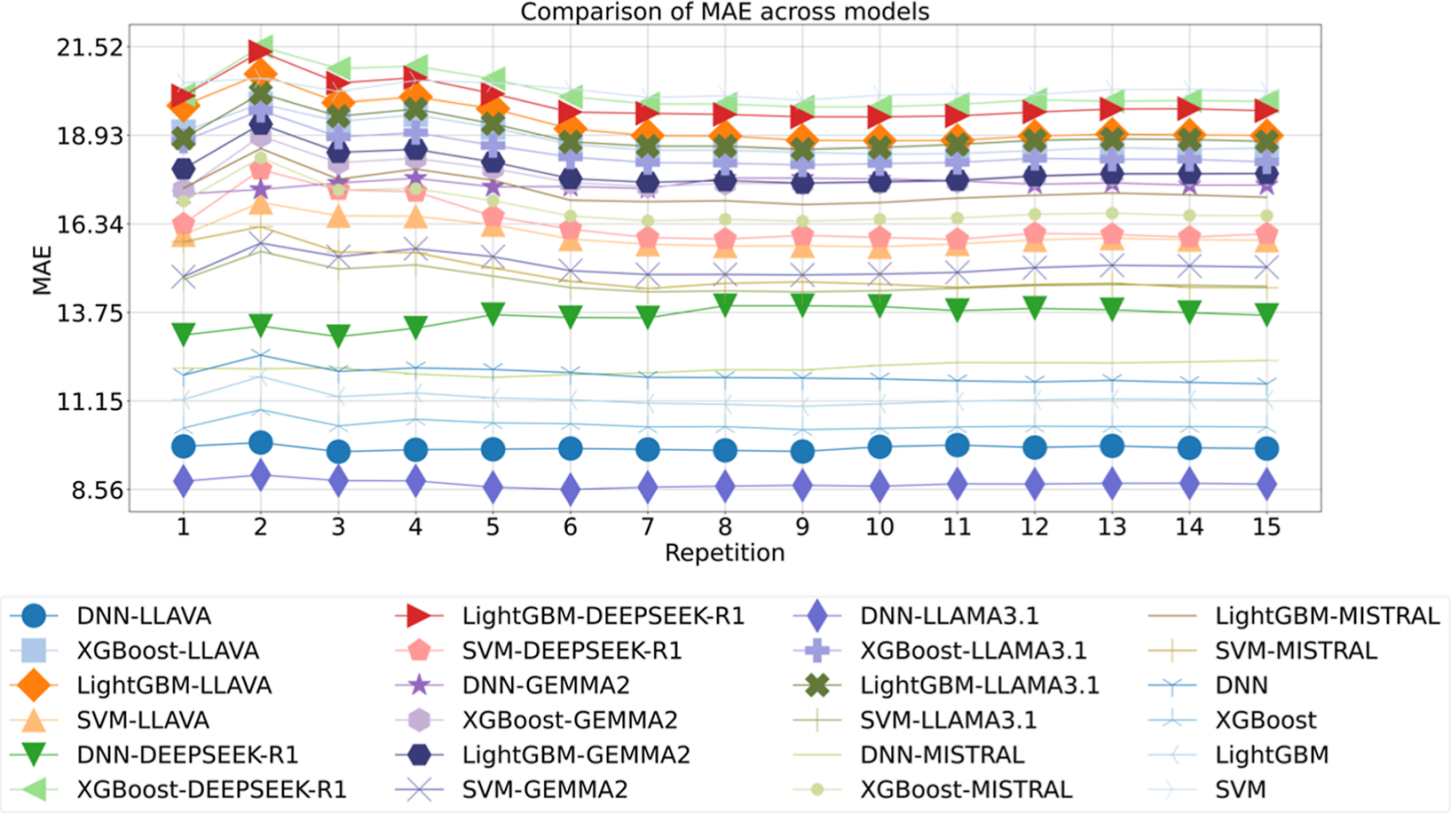
Comparison of MAE across models for droplet diameter of 1st data set, with the repetition label indicating iterations of 10-fold cross-validation.

To demonstrate the minimal standard error and the stabilization of mean estimated performance across metrics of droplet diameter prediction of first data set^25^ for different models, a comprehensive evaluation of various machine learning models combined with language models is provided. This evaluation focuses on MAE, MSE, RMSE, and *R* ^2^, along with their corresponding standard errors, as shown in Table 2.

**Table 2:** Metrics of Evaluation for Droplet Diameter Prediction of 1st Dataset, Compared Across Models

droplet diameter (μm)				
	metrics			
model	MAE	MSE	RMSE	*R* ^2^
DNN	11.652 ± 0.156	361.066 ± 20.284	19.546 ± 0.722	0.898 ± 0.01
DNN-DEEPSEEK-R1	13.081 ± 1.011	286.232 ± 14.594	17.236 ± 0.295	0.928 ± 0.008
DNN-GEMMA2	17.535 ± 0.6	593.202 ± 48.292	23.907 ± 1.238	0.856 ± 0.01
DNN-LLAMA3.1	8.991 ± 0.285	185.93 ± 17.79	13.317 ± 0.655	0.953 ± 0.004
DNN-LLAVA	9.764 ± 0.238	202.866 ± 10.56	14.018 ± 0.241	0.95 ± 0.006
DNN-MISTRAL	11.939 ± 0.288	298.655 ± 35.837	17.109 ± 0.239	0.916 ± 0.003
LightGBM	11.087 ± 0.295	382.905 ± 17.898	18.92 ± 0.438	0.894 ± 0.01
LightGBM-DEEPSEEK-R1	19.644 ± 0.262	1029.504 ± 48.572	34.482 ± 2.171	0.734 ± 0.009
LightGBM-GEMMA2	19.255 ± 0.703	837.197 ± 38.61	28.967 ± 2.392	0.784 ± 0.006
LightGBM-LLAMA3.1	18.658 ± 0.307	939.263 ± 53.154	31.019 ± 1.486	0.756 ± 0.008
LightGBM-LLAVA	18.78 ± 0.357	951.237 ± 36.212	31.009 ± 2.757	0.757 ± 0.006
LightGBM-MISTRAL	17.186 ± 0.292	941.377 ± 85.144	29.951 ± 3.239	0.773 ± 0.029
SVM	20.262 ± 0.309	1251.815 ± 193.003	32.737 ± 1.15	0.692 ± 0.033
SVM-DEEPSEEK-R1	16.336 ± 0.568	864.198 ± 191.984	28.074 ± 2.758	0.787 ± 0.022
SVM-GEMMA2	14.79 ± 0.957	627.973 ± 125.128	24.028 ± 2.25	0.838 ± 0.008
SVM-LLAMA3.1	14.343 ± 0.244	593.508 ± 24.529	23.798 ± 0.497	0.844 ± 0.005
SVM-LLAVA	16.56 ± 0.441	691.318 ± 37.424	25.515 ± 0.635	0.816 ± 0.005
SVM-MISTRAL	14.653 ± 0.362	724.282 ± 45.335	25.676 ± 0.682	0.795 ± 0.016
XGBoost	10.515 ± 0.291	330.289 ± 30.482	16.954 ± 0.436	0.923 ± 0.005
XGBoost-DEEPSEEK-R1	20.114 ± 1.021	1096.525 ± 125.638	32.106 ± 1.813	0.748 ± 0.015
XGBoost-GEMMA2	18.258 ± 0.449	713.531 ± 115.793	25.989 ± 1.952	0.807 ± 0.006
XGBoost-LLAMA3.1	18.066 ± 0.283	854.729 ± 70.847	28.401 ± 0.874	0.786 ± 0.011
XGBoost-LLAVA	19.526 ± 0.442	761.209 ± 99.043	27.546 ± 0.49	0.79 ± 0.005
XGBoost-MISTRAL	16.417 ± 0.307	716.579 ± 30.872	26.747 ± 0.964	0.816 ± 0.007

While the base DNN model performs well (MAE = 11.652 ; *R* ^2^ = 0.898), the integration of LLAMA3.1 further enhances performance, reducing MAE to 8.991, approximately a 10% reduction relative to the MAE of 10 reported in a prior study,^25^ along with a lower MSE of 185.93, RMSE of 13.317, and the highest *R* ^2^ of 0.953 among all models evaluated. Conversely, integrating foundation models into LightGBM, SVM, and XGBoost yields inconsistent effects. For instance, LightGBM’s performance markedly deteriorates when paired with DEEPSEEK-R1 (MAE = 19.644; *R* ^2^ = 0.734), compared to the base LightGBM model (MAE = 11.087; *R* ^2^ = 0.894). Similar regressions are seen in XGBoost-DEEPSEEK-R1 (MAE = 20.114; *R* ^2^ = 0.748) and other augmented variants, with MAE values exceeding 18 and *R* ^2^ dropping below 0.8 in most cases. Notably, all XGBoost and LightGBM combinations with LLAMA3.1, LLAVA, or MISTRAL underperform relative to their unmodified counterparts, suggesting these tree-based methods may be ill-suited to leverage LLM-derived embeddings for this regression task. In the SVM group, augmentation yields moderate gains in some cases. SVM-GEMMA2 and SVM-LLAMA3.1 improve upon base SVM performance (MAE reduced from 20.262 to 14.79 and 14.343, respectively), alongside increased *R* ^2^ values (0.838 and 0.844 versus 0.692). However, the standard errors in MSE and RMSE remain relatively high. Overall, DNN-LLAMA3.1 emerges as the most robust and accurate model across all metrics, suggesting that LLAMA3.1 embeddings may capture latent representations that align closely with the target variable in this regression setting. In contrast, tree-based models (LightGBM and XGBoost) appear less capable of integrating such embeddings effectively. These findings advocate for the use of deep neural architectures when leveraging LLM outputs in regression tasks.

To assess the trends in accuracy in droplet diameter prediction of second data set^42^ across different models, a detailed comparison of 24 models over 15 repetitions was conducted, using RMSE as the performance metric, as illustrated in Figure 4.

**Figure 4:**
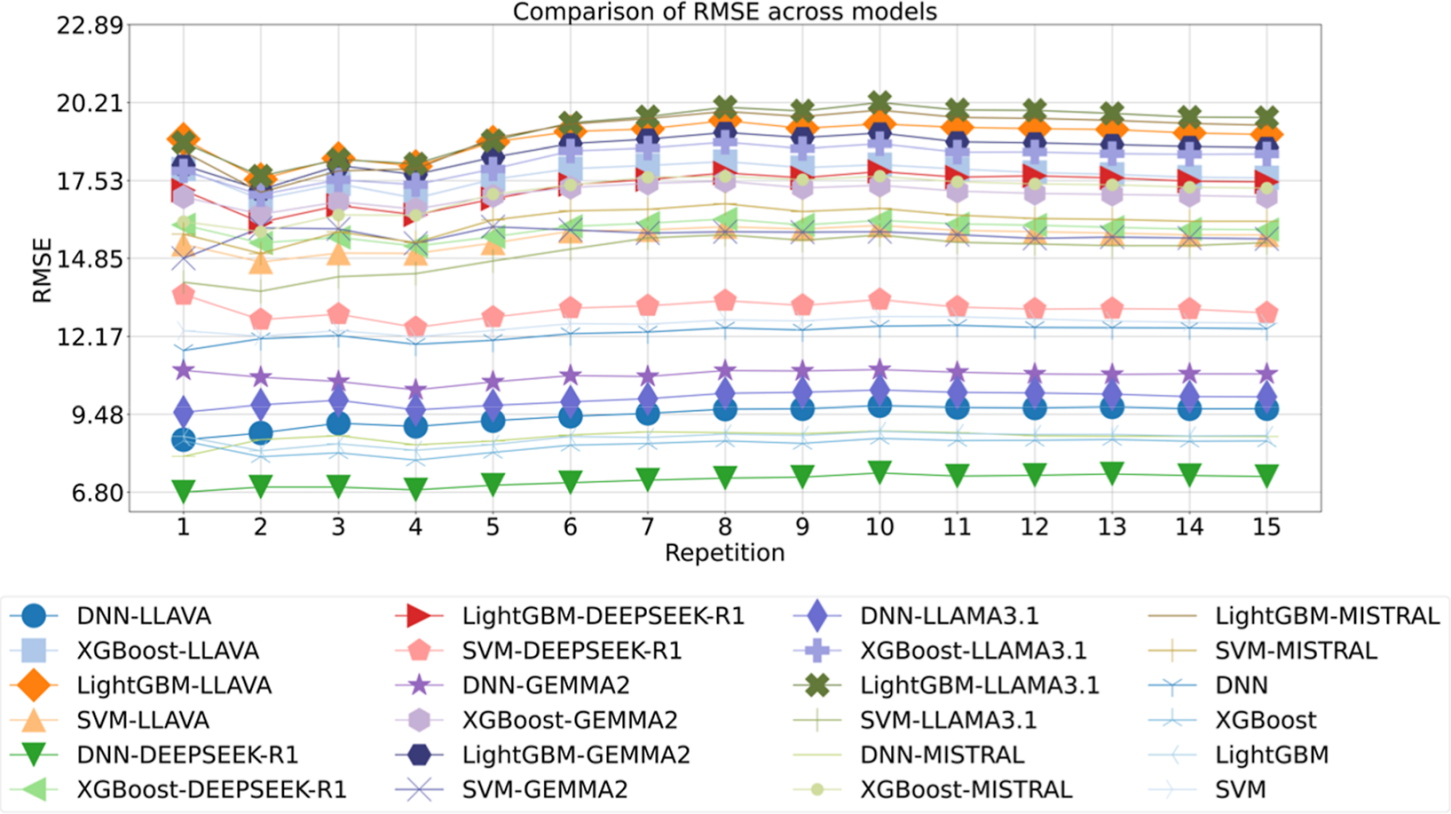
Comparison of RMSE across models for droplet diameter of 2nd data set, with the repetition label indicating iterations of 10-fold cross-validation.

Among all configurations, **DNN-DEEPSEEK-R1 achieved the lowest RMSE of around 6.80**, representing a substantial reduction compared to the baseline DNN model. Similarly, models enhanced with LLAVA embeddings outperformed baselines, with DNN-LLAVA yielding an RMSE of approximately 9.6 versus around 12.5 for DNN. These findings underscore the critical influence of embedding source on downstream model performance.

To demonstrate the minimal standard error and the stabilization of mean estimated performance across metrics of droplet diameter prediction of second data set^42^ for different models, a comprehensive evaluation of various machine learning models combined with language models is provided. This evaluation focuses on MAE, MSE, RMSE, and *R* ^2^, along with their corresponding standard errors, as shown in Table 3. Across all configurations, the baseline DNN combined with DEEPSEEK-R1 exhibits the most favorable overall performance, achieving the lowest MAE of 4.615, MSE of 57.543, and RMSE of 7.339, approximately a 26% reduction relative to the RMSE of 9.88 reported in a prior study,^42^ alongside the highest *R* ^2^ of 0.975, indicating superior predictive accuracy and generalizability.

**Table 3:** Metrics of Evaluation for Droplet Diameter Prediction of 2nd Dataset, Compared Across Models

droplet diameter (μm)				
	metrics			
model	MAE	MSE	RMSE	*R* ^2^
DNN	8.738 ± 0.141	157.28 ± 5.436	12.514 ± 0.203	0.925 ± 0.008
DNN-DEEPSEEK-R1	4.615 ± 0.187	57.543 ± 2.068	7.339 ± 0.128	0.975 ± 0.002
DNN-GEMMA2	7.071 ± 0.137	119.026 ± 8.646	10.78 ± 0.27	0.933 ± 0.008
DNN-LLAMA3.1	6.73 ± 0.106	106.497 ± 4.01	10.081 ± 0.18	0.94 ± 0.003
DNN-LLAVA	6.28 ± 0.102	88.774 ± 4.441	9.662 ± 0.204	0.959 ± 0.004
DNN-MISTRAL	5.093 ± 0.25	66.521 ± 7.096	8.613 ± 0.453	0.963 ± 0.005
LightGBM	5.11 ± 0.114	72.253 ± 5.801	8.898 ± 0.208	0.957 ± 0.001
LightGBM-DEEPSEEK-R1	9.532 ± 0.775	333.263 ± 22.599	17.56 ± 0.597	0.831 ± 0.007
LightGBM-GEMMA2	10.732 ± 0.299	350.412 ± 55.745	18.041 ± 0.665	0.807 ± 0.006
LightGBM-LLAMA3.1	10.49 ± 0.36	419.971 ± 19.647	19.695 ± 0.462	0.811 ± 0.026
LightGBM-LLAVA	11.42 ± 0.237	395.872 ± 74.03	18.949 ± 1.919	0.799 ± 0.006
LightGBM-MISTRAL	10.976 ± 0.338	379.597 ± 69.338	18.98 ± 0.831	0.811 ± 0.012
SVM	7.866 ± 0.145	152.954 ± 9.593	12.356 ± 0.876	0.908 ± 0.003
SVM-DEEPSEEK-R1	7.986 ± 0.178	193.468 ± 16.645	13.231 ± 0.452	0.904 ± 0.005
SVM-GEMMA2	8.989 ± 0.261	237.329 ± 41.812	15.747 ± 0.492	0.873 ± 0.022
SVM-LLAMA3.1	9.924 ± 0.196	249.098 ± 12.404	15.548 ± 0.52	0.873 ± 0.008
SVM-LLAVA	10.39 ± 0.207	264.182 ± 10.201	15.98 ± 0.297	0.876 ± 0.007
SVM-MISTRAL	9.371 ± 0.174	288.385 ± 15.732	16.334 ± 0.443	0.855 ± 0.005
XGBoost	4.509 ± 0.154	80.204 ± 4.165	8.655 ± 0.23	0.959 ± 0.004
XGBoost-DEEPSEEK-R1	9.742 ± 0.23	252.535 ± 30.858	15.376 ± 0.898	0.855 ± 0.009
XGBoost-GEMMA2	10.782 ± 0.193	291.414 ± 20.084	16.795 ± 0.558	0.837 ± 0.005
XGBoost-LLAMA3.1	10.802 ± 0.399	325.739 ± 30.363	17.552 ± 0.767	0.816 ± 0.011
XGBoost-LLAVA	10.61 ± 0.317	354.872 ± 68.804	17.804 ± 0.419	0.828 ± 0.004
XGBoost-MISTRAL	10.035 ± 0.319	330.642 ± 15.439	17.667 ± 0.484	0.842 ± 0.013

In contrast, the same DEEPSEEK-R1 augmentation leads to markedly worse performance when paired with LightGBM. Similar degradation patterns are observed for other LLM-augmented LightGBM and SVM models, suggesting that the integration of LLM outputs is beneficial primarily when coupled with DNN architectures. Interestingly, among the unaugmented models, XGBoost demonstrates strong baseline performance (MAE: 4.509; *R* ^2^: 0.959), comparable to DNN-MISTRAL (MAE: 5.093; *R* ^2^: 0.963), yet its performance declines substantially upon integration with any LLM variant. These findings underscore that the synergistic gains from combining LLMs with machine learning models are architecture-dependent, with DNNs uniquely capable of leveraging LLM-derived features to enhance predictive precision.

### Performance in Prediction of Droplet Generation Rate

To evaluate the trends in accuracy in droplet generation rate prediction of first data set^25^ across various models, a thorough comparison of the 24 models over 15 repetitions was carried out, utilizing MAE as the performance metric, as depicted in Figure 5.

**Figure 5:**
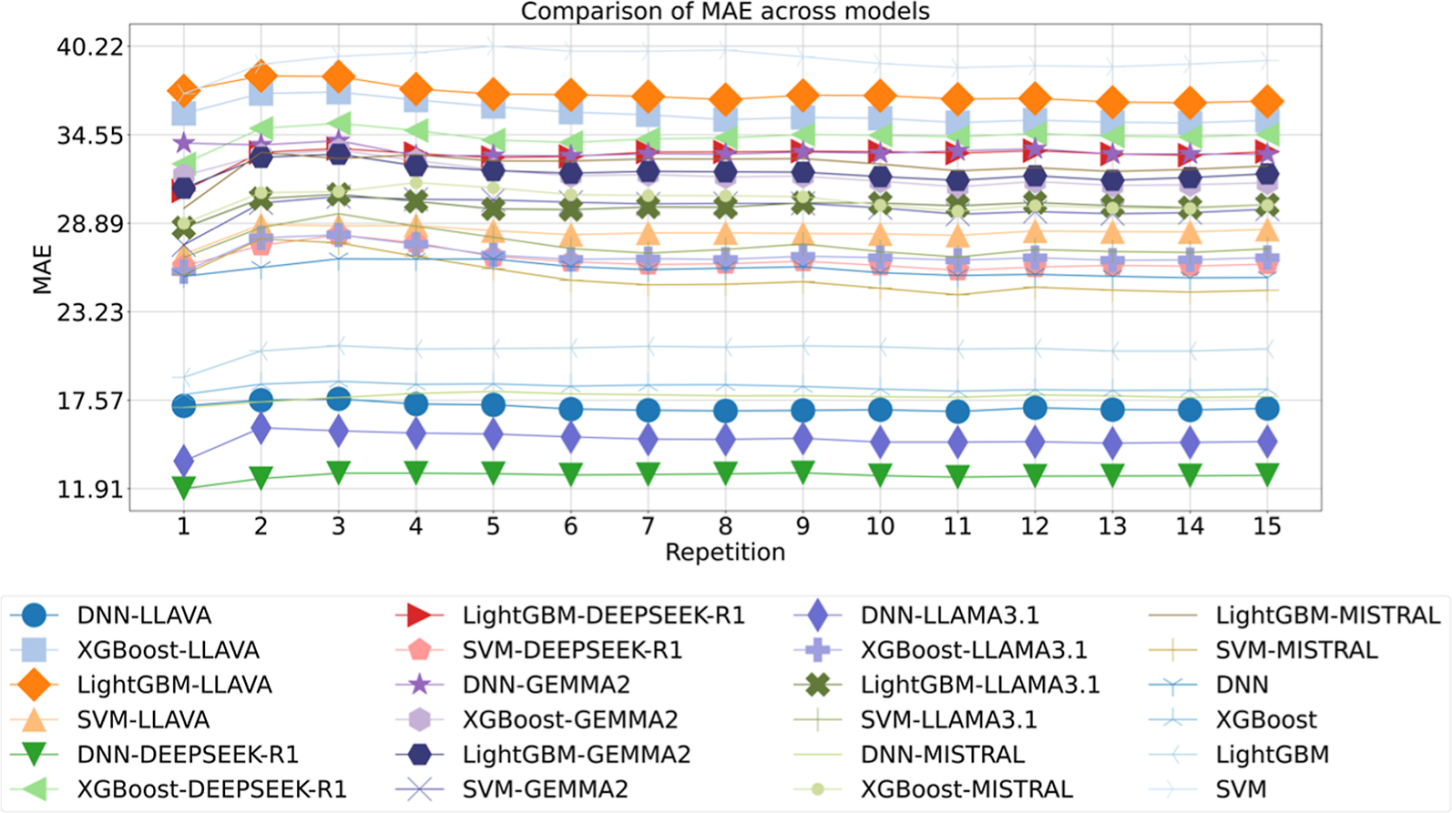
Comparison of MAE across models for droplet generation rate of 1st data set, with the repetition label indicating iterations of 10-fold cross-validation.

The figure demonstrates consistent performance trends, with the DNN-DEEPSEEK-R1 model achieving the lowest MAE across all repetitions, averaging approximately 11.91, indicating a superior predictive accuracy. In contrast, LightGBM-LLAVA exhibits the MAE, averaging near 37.4, representing an around 3.1-fold increase in error compared to the best-performing model. These results highlight the critical role of model architecture selection in predictive accuracy, particularly emphasizing the effectiveness of DNNs when paired with LLMs such as DEEPSEEK-R1. The findings underscore the need for rigorous benchmarking of downstream task performance when integrating LLMs with machine learning pipelines. To illustrate the minimal standard error and the stabilization of mean estimated performance across metrics of droplet generation rate prediction of first data set^25^ for different models, we present a comprehensive evaluation of the various machine learning models integrated with LLMs. This assessment again uses MAE, MSE, RMSE, and *R* ^2^, along with their associated standard errors, as presented in Table 4.

**Table 4:** Metrics of Evaluation for Droplet Generation Rate of 1st Dataset, Compared Across Models

	droplet generation rate (Hz)			
	metrics			
model	MAE	MSE	RMSE	*R* ^2^
DNN	26.09 ± 0.565	1491.512 ± 184.482	40.528 ± 1.597	0.878 ± 0.015
DNN-DEEPSEEK-R1	12.548 ± 0.597	493.782 ± 34.594	21.036 ± 0.683	0.967 ± 0.002
DNN-GEMMA2	33.336 ± 0.815	3128.157 ± 121.883	54.702 ± 1.064	0.804 ± 0.005
DNN-LLAMA3.1	14.881 ± 0.309	602.711 ± 153.99	25.059 ± 1.401	0.952 ± 0.004
DNN-LLAVA	17.65 ± 0.673	1006.409 ± 86.643	30.156 ± 1.272	0.935 ± 0.004
DNN-MISTRAL	17.846 ± 0.413	856.897 ± 110.924	28.29 ± 1.682	0.939 ± 0.007
LightGBM	21.044 ± 0.517	1631.712 ± 144.477	39.066 ± 2.367	0.914 ± 0.012
LightGBM-DEEPSEEK-R1	33.474 ± 0.598	3264.843 ± 331.256	59.595 ± 1.368	0.755 ± 0.008
LightGBM-GEMMA2	33.312 ± 1.034	3648.187 ± 261.725	60.54 ± 2.523	0.738 ± 0.028
LightGBM-LLAMA3.1	29.888 ± 0.552	3001.848 ± 308.985	57.175 ± 2.648	0.802 ± 0.008
LightGBM-LLAVA	36.799 ± 0.826	4617.506 ± 471.456	65.114 ± 1.423	0.722 ± 0.007
LightGBM-MISTRAL	32.248 ± 0.668	3246.93 ± 312.581	59.518 ± 2.016	0.762 ± 0.009
SVM	39.885 ± 0.798	4540.114 ± 206.091	65.615 ± 2.232	0.699 ± 0.014
SVM-DEEPSEEK-R1	26.133 ± 1.165	2730.758 ± 289.629	48.986 ± 2.349	0.787 ± 0.046
SVM-GEMMA2	30.159 ± 0.72	3269.004 ± 367.866	59.566 ± 1.745	0.747 ± 0.018
SVM-LLAMA3.1	27.983 ± 0.948	3013.179 ± 369.989	53.744 ± 2.624	0.781 ± 0.039
SVM-LLAVA	28.219 ± 0.606	2852.097 ± 303.784	50.91 ± 2.294	0.802 ± 0.026
SVM-MISTRAL	27.878 ± 1.727	3526.108 ± 535.563	57.406 ± 4.802	0.789 ± 0.02
XGBoost	18.237 ± 0.344	1180.476 ± 99.566	33.173 ± 1.447	0.923 ± 0.007
XGBoost-DEEPSEEK-R1	34.57 ± 0.506	3036.531 ± 266.357	54.588 ± 2.381	0.796 ± 0.021
XGBoost-GEMMA2	31.858 ± 0.589	2783.247 ± 113.693	52.684 ± 1.15	0.783 ± 0.017
XGBoost-LLAMA3.1	26.678 ± 0.424	1952.817 ± 175.142	43.705 ± 2.066	0.867 ± 0.016
XGBoost-LLAVA	36.769 ± 0.746	3451.831 ± 129.742	57.538 ± 1.085	0.768 ± 0.01
XGBoost-MISTRAL	28.875 ± 1.34	2856.466 ± 229.18	53.148 ± 2.112	0.81 ± 0.018

Among all configurations, DNN DEEPSEEK R1 delivers the strongest overall performance, achieving the lowest MAE of 12.548, approximately a 40% reduction relative to the MAE of 20 reported in a prior study,^25^ as well as lower MSE of 493.782 and RMSE of 21.036 while maintaining the highest *R* ^2^ of 0.967, indicating high predictive accuracy and consistency. In contrast, augmenting LightGBM with LLAVA yields the poorest performance, with an MAE of 36.799 and an *R* ^2^ of just 0.722.

Notably, base DNN and XGBoost models outperform their SVM and LightGBM counterparts in most cases, even prior to LLM augmentation. These findings highlight the critical importance of model-LLM alignment for downstream predictive modeling.

To assess the trends in accuracy in droplet generation rate prediction of second data set^42^ across different models, a detailed comparison of 24 models over 15 repetitions was conducted, using RMSE as the performance metric, as illustrated in Figure 6. The results reveal that **DNN-DEEPSEEK-R1** achieves the **lowest RMSE of around 462.3**, substantially outperforming all other combinations. In contrast, the baseline SVM model yields the highest RMSE of around 1106.7, indicating a performance improvement from best to worst. On average, embedding-enhanced models display marked reductions in RMSE compared to their nonaugmented counterparts. Critically, these findings underscore several important scientific implications. First, language model embeddings substantially improve predictive accuracy across diverse model architectures, suggesting that semantic features extracted from pretrained LLMs encapsulate domain-relevant structure not captured by traditional numerical features. Second, DNN models systematically benefit more from embedding integration, particularly with DEEPSEEK-R1 and LLAMA3.1, indicating a synergistic interaction between deep feature learning and rich contextual representations.

**Figure 6:**
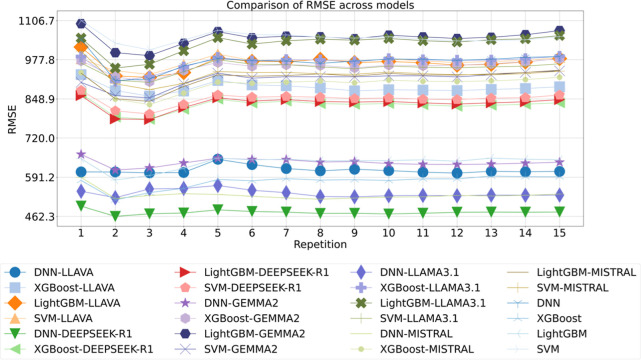
Comparison of RMSE across models for droplet generation rate of 2nd data set, with the repetition label indicating iterations of 10-fold cross-validation.

To illustrate the minimal standard error and the stabilization of mean estimated performance across metrics of droplet generation rate prediction of second data set^42^ for different models, we present a comprehensive evaluation of the various machine learning models integrated with LLMs. This assessment again uses MAE, MSE, RMSE, and *R* ^2^, along with their associated standard errors, as presented in Table 5. Among all configurations, **DNN-DEEPSEEK-R1** achieved the strongest overall performance, with the lowest MAE of 291.204, lowest RMSE of 476.428, approximately a 5% reduction relative to the RMSE of 499.9 reported in a prior study,^42^ and highest *R* ^2^ of 0.973, substantially outperforming the base DNN (MAE: 533.94, RMSE: 966.03, *R* ^2^: 0.88). **DNN-MISTRAL** and **DNN-LLAMA3.1** also demonstrated excellent predictive accuracy, both reaching an *R* ^2^ of 0.963, with RMSEs of 521.10 and 554.08 respectively. In contrast, hybrid models based on LightGBM, SVM, and XGBoost showed more modest improvements or even performance deterioration when paired with embeddings. For example, the LightGBM-GEMMA2 model showed a higher mean absolute error (511.92 compared to 261.26 in the base LightGBM) and a lower *R* ^2^ value (0.87 versus 0.955), reflecting a decline in model accuracy. Similarly, most SVM-based hybrids suffered in both absolute error and *R* ^2^ compared to their DNN counterparts. XGBoost and LightGBM base models independently achieved strong performance (e.g., XGBoost: *R* ^2^ = 0.957, RMSE = 581.81), highlighting their robustness; however, their integration with large language model embeddings did not yield consistent benefits, and in many cases introduced variance and error inflation. This disparity suggests that model-embedding synergy is highly architecture-dependent, with DNNs appearing better suited to leverage the semantic enrichment offered by foundation model embeddings. These findings underscore the potential of embedding-augmented deep learning models, particularly DNNs, in enhancing predictive accuracy in regression tasks.

**Table 5:** Metrics of Evaluation for Droplet Generation Rate of 2nd Dataset, Compared Across Models

	droplet generation rate (Hz)			
	Metrics			
model	MAE	MSE	RMSE	*R* ^2^
DNN	533.94 ± 22.125	872804.852 ± 92329.903	966.028 ± 25.966	0.88 ± 0.004
DNN-DEEPSEEK-R1	291.204 ± 10.128	222428.061 ± 19780.729	476.428 ± 9.307	0.973 ± 0.002
DNN-GEMMA2	398.514 ± 20.067	424771.441 ± 26662.35	640.565 ± 12.163	0.952 ± 0.004
DNN-LLAMA3.1	297.423 ± 9.509	335692.435 ± 33865.376	554.078 ± 26.781	0.963 ± 0.002
DNN-LLAVA	299.755 ± 20.771	406829.335 ± 71894.46	608.316 ± 53.922	0.956 ± 0.007
DNN-MISTRAL	282.942 ± 11.61	305421.128 ± 22724.034	521.101 ± 30.78	0.963 ± 0.003
LightGBM	261.259 ± 20.003	387727.036 ± 41868.643	596.654 ± 32.523	0.955 ± 0.006
LightGBM-DEEPSEEK-R1	443.073 ± 15.8	655797.061 ± 76065.107	781.197 ± 34.528	0.922 ± 0.005
LightGBM-GEMMA2	511.921 ± 31.949	1141204.358 ± 93468.156	1001.148 ± 70.894	0.87 ± 0.015
LightGBM-LLAMA3.1	526.546 ± 21.529	1163101.911 ± 170595.136	1049.428 ± 78.615	0.86 ± 0.005
LightGBM-LLAVA	452.224 ± 20.352	914204.946 ± 108203.501	919.859 ± 47.249	0.88 ± 0.018
LightGBM-MISTRAL	419.585 ± 20.076	916144.612 ± 133550.668	925.408 ± 27.076	0.909 ± 0.008
SVM	649.875 ± 25.317	1106864.868 ± 88690.828	1042.445 ± 34.698	0.866 ± 0.006
SVM-DEEPSEEK-R1	438.459 ± 16.759	795415.918 ± 57864.165	857.005 ± 26.385	0.909 ± 0.005
SVM-GEMMA2	450.896 ± 18.547	896491.552 ± 58587.635	852.197 ± 37.264	0.898 ± 0.006
SVM-LLAMA3.1	539.009 ± 39.721	891209.348 ± 99056.246	910.516 ± 55.754	0.893 ± 0.011
SVM-LLAVA	555.533 ± 18.885	933166.886 ± 86640.296	980.792 ± 56.383	0.879 ± 0.006
SVM-MISTRAL	504.24 ± 44.6	906958.237 ± 140313.385	898.659 ± 70.488	0.895 ± 0.014
XGBoost	268.668 ± 6.533	371440.978 ± 26306.438	581.812 ± 20.29	0.957 ± 0.003
XGBoost-DEEPSEEK-R1	470.348 ± 36.296	787914.936 ± 105530.811	848.219 ± 29.339	0.922 ± 0.005
XGBoost-GEMMA2	527.151 ± 12.35	916520.047 ± 106346.316	942.793 ± 37.02	0.888 ± 0.007
XGBoost-LLAMA3.1	551.577 ± 14.867	876401.62 ± 94850.479	978.13 ± 27.434	0.89 ± 0.008
XGBoost-LLAVA	448.774 ± 17.837	898476.778 ± 114520.592	884.794 ± 22.516	0.904 ± 0.007
XGBoost-MISTRAL	466.049 ± 17.76	793747.162 ± 57715.823	903.72 ± 24.016	0.895 ± 0.003

### Performance in Prediction of Droplet Regime

To evaluate the trends in accuracy in droplet regime predictions of first data set^25^ across various models, a detailed comparison of 24 models across 15 repetitions was performed, with accuracy serving as the primary performance metric, as shown in Figure 7. Notably, the DNN-LLAMA3.1 model consistently outperforms all other configurations, achieving a peak accuracy of about 0.982 on the first repetition and maintaining a stable performance around 0.973 across subsequent trials. In contrast, models leveraging DEEPSEEK-R1 exhibit the lowest performance, with XGBoost-DEEPSEEK-R1 stabilizing near 0.938, representing an around 4.4% absolute decrease in accuracy compared to DNN-LLAMA3.1.

**Figure 7:**
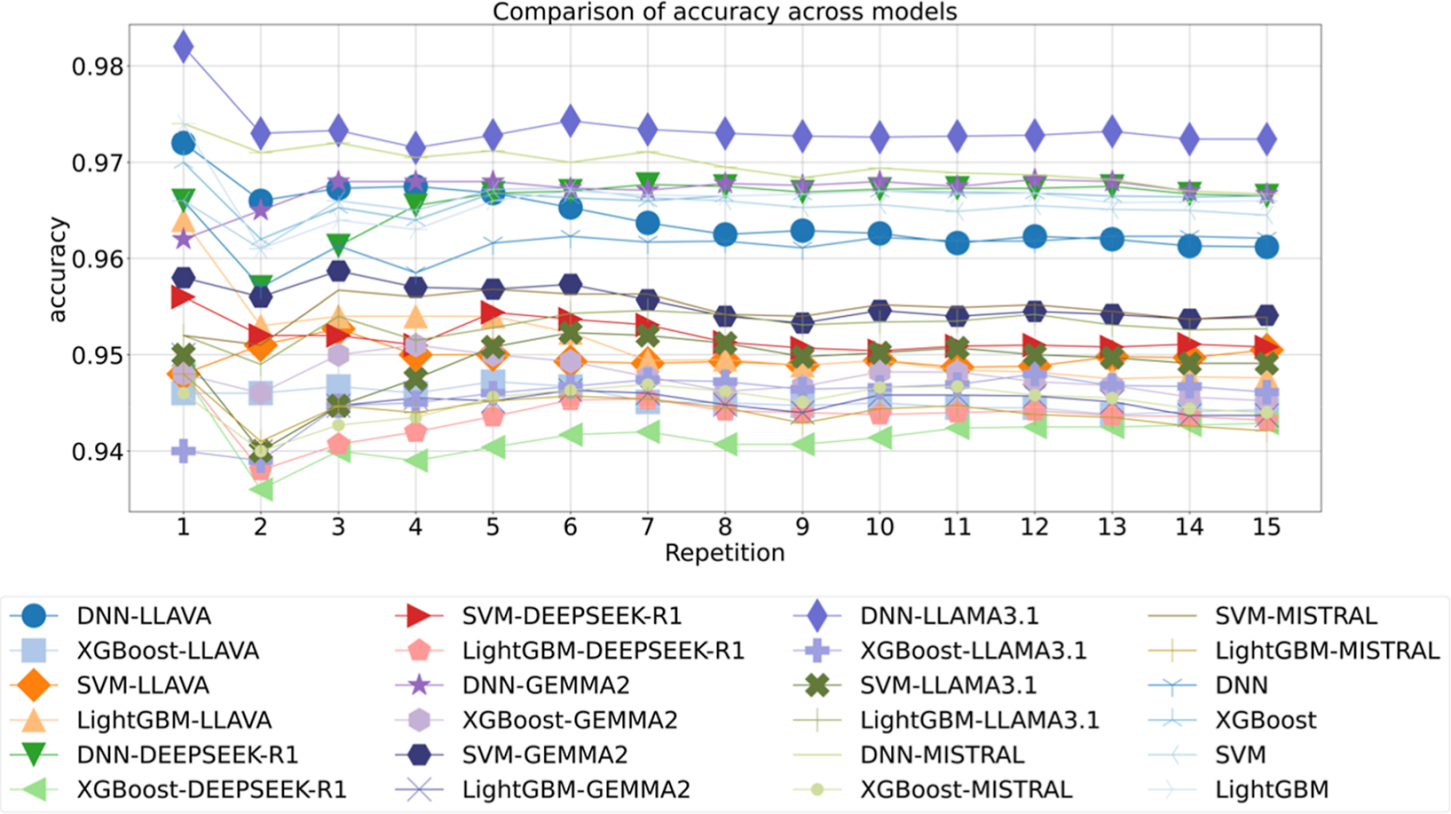
Comparison of accuracy across models for droplet regime of 1st data set, with the repetition label indicating iterations of 10-fold cross-validation.

Intermediate performance tiers are occupied by models based on GEMMA2 and MISTRAL, with DNN-GEMMA2 and SVM-GEMMA2 maintaining accuracies between 0.962 and 0.966. These findings suggest that the choice of both LLMs and downstream classifier exerts a substantial impact on predictive fidelity, with LLAMA3.1 representations being particularly amenable to deep learning architectures. To demonstrate the minimal standard error and the stabilization of mean estimated performance across metrics of droplet regime prediction of first data set^25^ for various models, we present an extensive comparative analysis of DNNs, LightGBM, SVM, and XGBoost. The performance of each model was evaluated when integrated with advanced LLMs, as outlined in Table 6. Key metrics of accuracy, F1 score, precision, recall, and ROC AUC are employed to examine the efficacy of these models. The table reports values obtained from the repetitions in which the difference between the mean and median of the corresponding metric was minimized. **DNN-LLAMA3.1** exhibits superior performance across nearly all metrics, with accuracy reaching 0.982, representing an improvement of more than 3% over the accuracy of 0.951 reported in a previous study,^25^ and ROC AUC peaking at 0.982, suggesting robust predictive capacity and class separability. Its F1-score of 0.979 and precision of 0.981 both exceed those of the baseline DNN (F1:0.955; precision: 0.972), implying a more balanced and accurate classification, while recall remains comparably high (0.966 vs 0.961).

**Table 6:** Metrics of Evaluation for Droplet Regime of 1st Dataset, Compared Across Models

	droplet regime				
	metrics				
model	accuracy	F1 score	precision	recall	ROC AUC
DNN	0.961 ± 0.003	0.955 ± 0.004	0.972 ± 0.006	0.961 ± 0.013	0.959 ± 0.004
DNN-DEEPSEEK-R1	0.961 ± 0.005	0.963 ± 0.003	0.959 ± 0.007	0.96 ± 0.004	0.961 ± 0.005
DNN-GEMMA2	0.968 ± 0.002	0.963 ± 0.003	0.961 ± 0.009	0.956 ± 0.006	0.966 ± 0.003
DNN-LLAMA3.1	0.982 ± 0.005	0.979 ± 0.006	0.981 ± 0.009	0.966 ± 0.003	0.982 ± 0.006
DNN-LLAVA	0.961 ± 0.002	0.958 ± 0.003	0.964 ± 0.003	0.954 ± 0.007	0.962 ± 0.002
DNN-MISTRAL	0.97 ± 0.004	0.966 ± 0.004	0.976 ± 0.005	0.957 ± 0.004	0.966 ± 0.002
LightGBM	0.961 ± 0.006	0.962 ± 0.003	0.976 ± 0.005	0.959 ± 0.012	0.964 ± 0.002
LightGBM-DEEPSEEK-R1	0.938 ± 0.008	0.938 ± 0.004	0.951 ± 0.013	0.935 ± 0.011	0.945 ± 0.004
LightGBM-GEMMA2	0.95 ± 0.011	0.936 ± 0.004	0.956 ± 0.005	0.924 ± 0.005	0.942 ± 0.003
LightGBM-LLAMA3.1	0.952 ± 0.006	0.948 ± 0.003	0.953 ± 0.007	0.934 ± 0.01	0.951 ± 0.007
LightGBM-LLAVA	0.949 ± 0.004	0.942 ± 0.004	0.963 ± 0.003	0.92 ± 0.004	0.947 ± 0.004
LightGBM-MISTRAL	0.948 ± 0.009	0.943 ± 0.01	0.961 ± 0.011	0.913 ± 0.01	0.942 ± 0.003
SVM	0.961 ± 0.004	0.96 ± 0.002	0.973 ± 0.009	0.952 ± 0.012	0.963 ± 0.004
SVM-DEEPSEEK-R1	0.956 ± 0.006	0.948 ± 0.003	0.953 ± 0.008	0.954 ± 0.012	0.956 ± 0.006
SVM-GEMMA2	0.959 ± 0.005	0.953 ± 0.006	0.955 ± 0.006	0.953 ± 0.011	0.958 ± 0.005
SVM-LLAMA3.1	0.94 ± 0.008	0.945 ± 0.006	0.959 ± 0.007	0.911 ± 0.015	0.949 ± 0.006
SVM-LLAVA	0.951 ± 0.007	0.943 ± 0.005	0.936 ± 0.014	0.953 ± 0.004	0.95 ± 0.007
SVM-MISTRAL	0.957 ± 0.004	0.95 ± 0.004	0.956 ± 0.006	0.951 ± 0.008	0.956 ± 0.006
XGBoost	0.97 ± 0.006	0.962 ± 0.002	0.978 ± 0.007	0.956 ± 0.012	0.96 ± 0.006
XGBoost-DEEPSEEK-R1	0.94 ± 0.006	0.936 ± 0.003	0.941 ± 0.004	0.944 ± 0.012	0.942 ± 0.004
XGBoost-GEMMA2	0.95 ± 0.005	0.938 ± 0.007	0.957 ± 0.004	0.922 ± 0.006	0.944 ± 0.003
XGBoost-LLAMA3.1	0.94 ± 0.01	0.937 ± 0.006	0.936 ± 0.012	0.934 ± 0.015	0.946 ± 0.003
XGBoost-LLAVA	0.944 ± 0.003	0.935 ± 0.003	0.978 ± 0.006	0.907 ± 0.012	0.94 ± 0.003
XGBoost-MISTRAL	0.94 ± 0.007	0.938 ± 0.004	0.961 ± 0.011	0.917 ± 0.006	0.942 ± 0.003

These improvements are particularly noteworthy given the low standard error values, reflecting high statistical confidence. Similarly, **DNN-MISTRAL** and **DNN-GEMMA2** show consistent gains in performance, underscoring that certain LLM embeddings can meaningfully enrich feature representations when paired with deep architectures. In contrast, **LightGBM** and **XGBoost** models show reduced responsiveness to LLM embeddings.

### Enhanced Efficiency for Design Automation

For example, **LightGBM-DEEPSEEK-R1** suffers a decline in accuracy of 0.938 and recall of 0.935 compared to the baseline LightGBM (0.961 and 0.959, respectively), with an absolute drop of 2.3% in accuracy. Moreover, **LightGBM-MISTRAL** and **XGBoost-MISTRAL** both exhibit significant declines in recall (0.913 and 0.917, respectively). SVM performance remains largely stable across LLM variants, with modest gains or losses. However, **SVM-LLAMA3.1** notably underperforms, with a 2.1% drop in accuracy of 0.940 and a pronounced decrease in recall of 0.911 relative to the baseline SVM (0.961 and 0.952), suggesting poor integration of LLAMA3.1 features with the SVM’s hyperplane-based decision boundaries. This mismatch highlights the importance of embedding-model compatibility; powerful embeddings may not yield improvements if their representational properties do not align with the downstream classifier’s decision mechanism. To analyze the trends in accuracy in droplet capillary number of first data set^25^ predictions across various models, a detailed comparison of 24 models was conducted over 15 repetitions, employing RMSE as the performance metric, as shown in Figure 8. The lowest RMSE of around 0.139 was achieved by DNN-GEMMA2, followed closely by DNN-LLAVA at around 0.16, indicating a clear advantage for DNN models when enriched with semantic embeddings. In contrast, the highest RMSE was observed in SVM-LLAVA and DEEPSEEK-R1 at around 0.18 and 0.21, respectively. These findings underscore the importance of model-embedding compatibility in yielding superior downstream performance.

**Figure 8:**
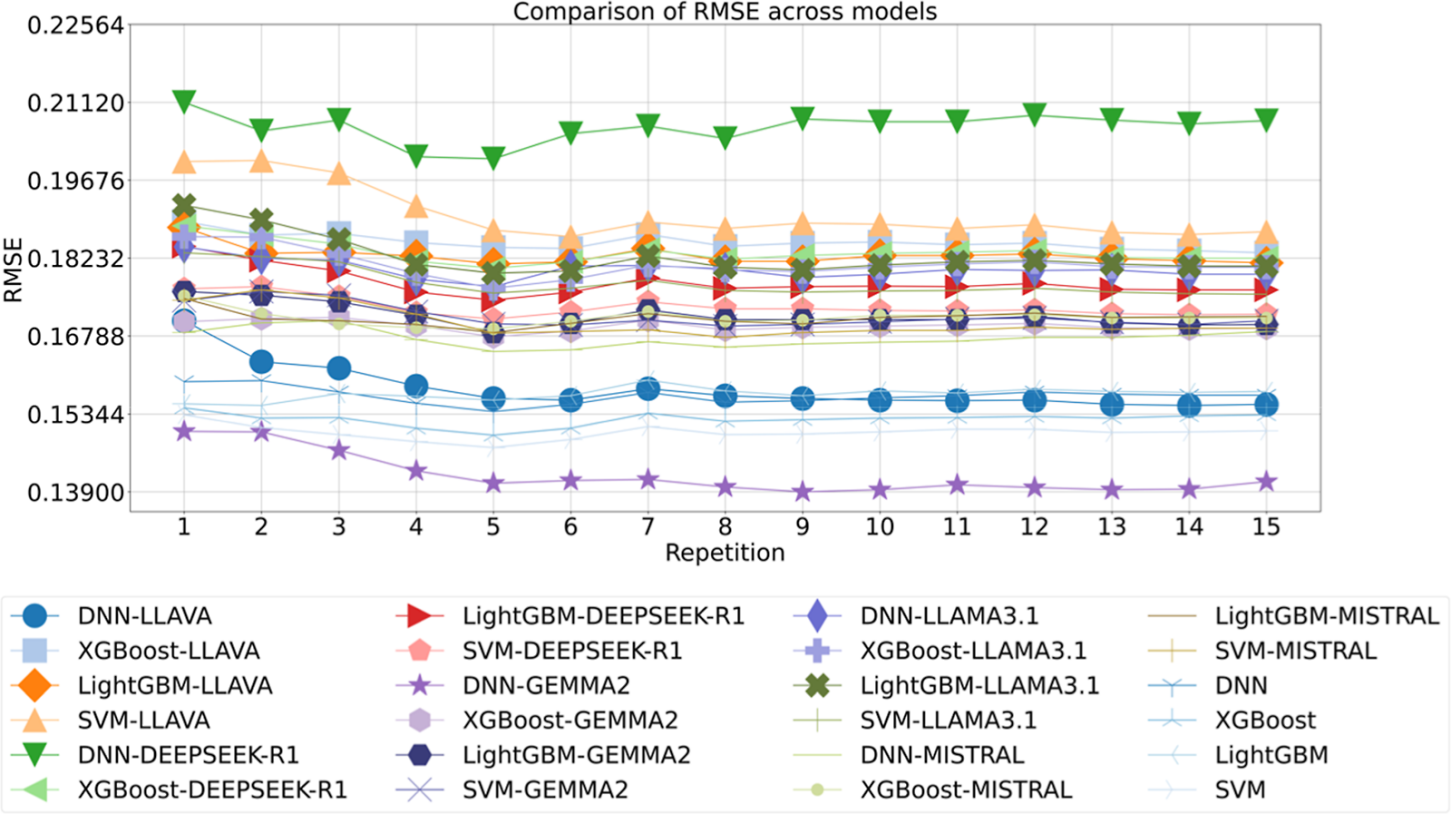
Comparison of RMSE across models for droplet capillary number of 1st data set, with the repetition label indicating iterations of 10-fold cross-validation.

To highlight the low standard error and the stabilization of mean estimation performance across metrics of prediction for droplet capillary number for different models, we present an extensive comparative analysis of DNN, LightGBM, SVM, and XGBoost paired with the various LLMs, as detailed in Table 7. Among all configurations, **DNN-GEMMA2** demonstrates the most robust performance, achieving the lowest MSE of 0.023, RMSE of 0.140, and the highest *R* ^2^ of 0.802, along with a strong MAE of 0.093. Similarly, **SVM and XGBoost** baselines perform competitively, with SVM achieving an MAE of 0.094, RMSE of 0.150, and *R* ^2^ of 0.785, while XGBoost exhibits near-identical performance (MAE 0.095; RMSE 0.153; *R* ^2^ 0.782). **DNN-LLAVA** achieves a low MAE of 0.093, moderate RMSE of 0.156, and an *R* ^2^ of 0.761. In contrast, **DNN-DEEPSEEK-R1** suffers a 42% increase in MAE of 0.149 and a 28% increase in RMSE of 0.201 compared to the DNN baseline, with a substantial decline in *R* ^2^ to 0.585 ± 0.023. Similarly, **LightGBM-LLAMA3.1** records one of the highest RMSE values of 0.192 and lowest *R* ^2^ of 0.692 among the LightGBM variants. Overall, the superior performance of DNN-GEMMA2 highlights the potential of targeted embedding-model pairings, but also emphasizes the necessity for careful embedding selection and feature integration strategies to avoid performance degradation.

**Table 7:** Metrics of Evaluation for Droplet Capillary Number of 1st Dataset, Compared Across Models

	droplet capillary number			
	metrics			
model	MAE	MSE	RMSE	*R* ^2^
DNN	0.105 ± 0.003	0.025 ± 0.001	0.154 ± 0.003	0.768 ± 0.014
DNN-DEEPSEEK-R1	0.149 ± 0.005	0.045 ± 0.002	0.201 ± 0.006	0.585 ± 0.023
DNN-GEMMA2	0.093 ± 0.002	0.023 ± 0.002	0.14 ± 0.003	0.802 ± 0.012
DNN-LLAMA3.1	0.129 ± 0.003	0.035 ± 0.003	0.177 ± 0.004	0.677 ± 0.015
DNN-LLAVA	0.093 ± 0.003	0.025 ± 0.001	0.156 ± 0.004	0.761 ± 0.012
DNN-MISTRAL	0.116 ± 0.002	0.029 ± 0.001	0.168 ± 0.002	0.737 ± 0.018
LightGBM	0.084 ± 0.002	0.026 ± 0.001	0.158 ± 0.004	0.779 ± 0.019
LightGBM-DEEPSEEK-R1	0.117 ± 0.004	0.032 ± 0.001	0.178 ± 0.003	0.707 ± 0.006
LightGBM-GEMMA2	0.106 ± 0.005	0.031 ± 0.001	0.17 ± 0.004	0.726 ± 0.008
LightGBM-LLAMA3.1	0.112 ± 0.002	0.036 ± 0.002	0.192 ± 0.007	0.692 ± 0.007
LightGBM-LLAVA	0.114 ± 0.004	0.034 ± 0.001	0.183 ± 0.005	0.689 ± 0.009
LightGBM-MISTRAL	0.108 ± 0.006	0.03 ± 0.001	0.168 ± 0.004	0.723 ± 0.007
SVM	0.094 ± 0.003	0.022 ± 0.001	0.15 ± 0.003	0.785 ± 0.006
SVM-DEEPSEEK-R1	0.117 ± 0.002	0.03 ± 0.001	0.174 ± 0.003	0.72 ± 0.008
SVM-GEMMA2	0.124 ± 0.004	0.029 ± 0.001	0.17 ± 0.003	0.722 ± 0.011
SVM-LLAMA3.1	0.121 ± 0.002	0.032 ± 0.001	0.176 ± 0.004	0.706 ± 0.007
SVM-LLAVA	0.128 ± 0.002	0.036 ± 0.001	0.188 ± 0.005	0.658 ± 0.014
SVM-MISTRAL	0.116 ± 0.004	0.029 ± 0.001	0.169 ± 0.002	0.723 ± 0.008
XGBoost	0.095 ± 0.002	0.023 ± 0.001	0.153 ± 0.002	0.782 ± 0.013
XGBoost-DEEPSEEK-R1	0.124 ± 0.003	0.034 ± 0.001	0.183 ± 0.003	0.686 ± 0.006
XGBoost-GEMMA2	0.109 ± 0.004	0.029 ± 0.001	0.17 ± 0.003	0.73 ± 0.007
XGBoost-LLAMA3.1	0.119 ± 0.002	0.035 ± 0.002	0.186 ± 0.006	0.683 ± 0.02
XGBoost-LLAVA	0.123 ± 0.004	0.035 ± 0.001	0.184 ± 0.002	0.675 ± 0.019
XGBoost-MISTRAL	0.115 ± 0.006	0.03 ± 0.001	0.17 ± 0.004	0.721 ± 0.01

## Conclusion

In this paper, we present μ-Fluidic-LLMs, an innovative framework designed to process tabular data associated with droplet microfluidics by transforming it into a text-based representation that effectively incorporates essential contextual information, including column descriptions. Our findings underscore the critical role of model selection and LLM integration in achieving optimal efficiency of performance prediction and design automation. We emphasize the superior performance of DNN models, particularly when integrated with advanced LLMs such as LLAMA3.1 and DEEPSEEK-R1. In comparison, traditional ensemble methods like LightGBM and XGBoost, even when incorporating state-of-the-art LLMs, may struggle to achieve comparable performance levels.

Multiple research directions can be pursued to further advance and optimize our framework. For example, exploration of various text serialisation methods, regularisation techniques, data set augmentation, or advanced optimization algorithms could provide means to enhance model performance and reduce error rates. Moreover, μ-Fluidic-LLMs is a general framework that can be adapted for use with other microfluidic components, such as micromixers,^58^ and extended to nonmicrofluidic structures like 3D-printed lattices,^59^ thereby enabling the automation of intricate design processes. In addition, μ-Fluidic-LLMs can be seamlessly integrated with existing microfluidic computer-aided design tools^42,60–67^ to enable more sophisticated and advanced design automation. Furthermore, the integration of automation technologies with powerful LLMs will open the door to developing a sought-after system that autonomously designs and executes scientific experiments.^31,68–71^ We anticipate that this work will inspire further research into the application of language technologies across a broader range of microfluidic applications.

## Supplementary Material



## Data Availability

https://github.com/duydinhlab/MicrofluidicLLMs

## Notes

The key parameters for tuning models, Table S1. Code segment revised as reviewer’s suggestion, Table S2. Metrics report for validation data of droplet diameter (μm) of first data set. Table S3. Metrics report for validation data of droplet generation rate (Hz) of first data set, Table S5. Metrics report for validation data of droplet diameter (μm) of second data set, Table S6. Metrics report for validation data of droplet generation rate (Hz) of second data set, Table S7. Metrics report for validation data of droplet capillary number of first data set (PDF)
