# Supplementary material for: Autonomous Droplet Microfluidic Design Framework with Large Language Models
Source: ACS Omega. 2025 Sep 26;10(39):45801–14. doi: 10.1021/acsomega.5c06253 (PMC12508994; doi:10.1021/acsomega.5c06253)
Supplement: Supplementary file 1 [file ao5c06253_si_001.pdf]

Supporting Information

Autonomous Droplet Microfluidic Design Framework with Large Language Models

Dinh-Nguyen Nguyen[1], *Raymond Kai-Yu Tong*[1], *Ngoc-Duy Dinh*[1*]

[1]Department of Biomedical Engineering, The Chinese University of Hong Kong, Shatin, N.T., Hong Kong, China

*Corresponding Author

Corresponding Email: ngocduydinh@cuhk.edu.hk

Key parameters for tuning models

XGBOOST:

```
parameters = {
    'max_depth': np.arange(2, 20, step=1),
    'learning_rate': [0.01, 0.02, 0.015, 0.025, 0.03, 0.001, 0.005, 0.0001],
    'colsample_bytree': [0.6, 0.7, 0.8],
    'colsample_bylevel': np.arange(0.4, 1.0, 0.1),
    'n_estimators': np.arange(50, 500, step=50),
    'reg_alpha': np.arange(0.01, 1, step=0.01),
    'subsample': np.arange(0.3, 1.0, 0.1),
    'reg_lambda': np.arange(0.01, 1, step=0.01)
}
```

Optimization Algorithms: RandomizedSearchCV

Loss metrics: ‘reg:squarederror’for regression; 'binary:logistic' for classification

Evaluation metrics: ‘Root Mean Squared Error’ for regression, ‘logloss’ for classification

References:

https://xgboost.readthedocs.io/en/latest/python/index.html

https://scikit-learn.org/stable/modules/generated/sklearn.model_selection.RandomizedSearchCV.html

LightGBM:

parameters = {

'max_depth': np.arange(2, 50, step=1),

'learning_rate': [0.01, 0.001, 0.005, 0.0001],

'colsample_bytree': np.arange(0.1, 1.0, 0.1),

'n_estimators': np.arange(50, 2000, step=50),

'reg_alpha': np.arange(0.01, 1, step=0.01),

'subsample': np.arange(0.3, 1.0, 0.1),

'num_leaves': np.arange(10, 300, 10),

'reg_lambda': np.arange(0.01, 1, step=0.01)

}

Optimization Algorithms: RandomizedSearchCV

Loss function: 'Mean Absolute Error' for regression; 'binary' for classification

Evaluation metric: 'Root Mean Squared Error' for regression; 'binary_logloss' for classification

References:

https://lightgbm.readthedocs.io/en/latest/pythonapi/lightgbm.LGBMRegressor.html

https://scikit-learn.org/stable/modules/generated/sklearn.model_selection.RandomizedSearchCV.html

SVM:

parameters = {

'C': [0.1, 1, 10, 100],

'kernel': ['linear', 'rbf', 'poly', 'sigmoid'],

'gamma': [1, 0.1, 0.01, 0.001, 'scale', 'auto']

}

Optimization Algorithms: RandomizedSearchCV

Loss function: ‘ε-insensitive loss function’ for regression; ‘hinge loss’ for classification

Evaluation metric: ‘Mean Absolute Error’ for regression; ‘accuracy’ for classification

References:

https://scikit-learn.org/stable/modules/generated/sklearn.svm.SVR.html

https://scikit-learn.org/stable/modules/generated/sklearn.svm.SVC.html

https://scikit-learn.org/stable/modules/generated/sklearn.model_selection.RandomizedSearchCV.html

DNN:

Drop_out=[min_value=0.0, max_value=0.9, step=0.1]

Learning rate=[min_value=1e-5, max_value=1e-3, sampling="log"]

Optimizer: Adam algorithm

Loss function: ‘Mean Squared Error’ for regression; ‘sparse_categorical_crossentropy’ for classification

Evaluation Metrics: ‘Mean Absolute Error’ for regression; "accuracy" for classification

Hyperparam Tuning: BayesianOptimization Tuner

References:

https://keras.io/api/optimizers/adam/

https://keras.io/keras_tuner/api/tuners/bayesian/

Code segment revised as reviewer’s suggestion
**from tensorflow.keras.models import clone_model as clone_DNN**<br>**from sklearn.base import clone as clone_ML**<br><br>for repeat in range(n_repeats):<br>kf = KFold(n_splits=n_splits, shuffle=True, random_state=repeat)<br>for train_index, test_index in kf.split(X):<br><br>**# 80% train, 20% temp**<br>X_train, X_temp = X[train_index], X[test_index]<br>y_train, y_temp = y[train_index], y[test_index]

```
# 10% val, 10% test
X_val, X_test, y_val, y_test = train_test_split(
    X_temp, y_temp, test_size=0.5, random_state=repeat
)

if model_name in ['DNN-LLAVA','DNN-DEEPSEEK-R1', 'DNN-GEMMA2',
'DNN-LLAMA3.1', 'DNN-MISTRAL', 'DNN']:

    model = clone_DNN(model_func)
    model.set_weights(model_func.get_weights())
    model.compile(optimizer='adam', loss='mean_squared_error', metrics=['mae'])
    model.fit(X_train, y_train, validation_data=(X_val, y_val), epochs=EPOCH * 10,
            callbacks=[stop_early], shuffle=True, verbose=VERBOSE,
batch_size=BATCH_SIZE)



elif model_name in ['XGBoost', 'XGBoost-LLAVA', 'XGBoost-DEEPSEEK-R1',
'XGBoost-GEMMA2', 'XGBoost-LLAMA3.1', 'XGBoost-MISTRAL']:

    model = clone_ML(model_func)
    model.fit(X_train, y_train, eval_set=[(X_val, y_val)], verbose=False)

elif model_name in ['LightGBM', 'LightGBM-LLAVA', 'LightGBM-DEEPSEEK-
R1', 'LightGBM-GEMMA2', 'LightGBM-LLAMA3.1', 'LightGBM-MISTRAL']:

    model = clone_ML(model_func)
    model.fit(X_train, y_train, eval_set=[(X_val, y_val)])

elif model_name in ['SVM', 'SVM-LLAVA', 'SVM-DEEPSEEK-R1', 'SVM-
GEMMA2', 'SVM-LLAMA3.1', 'SVM-MISTRAL']:
```

```
model = clone_ML(model_func)
model.fit(X_train, y_train)
```

Table S1. Code segment revised as reviewer's suggestion

Validation data				
Model	Droplet Diameter (μm)			
	Metrics			
	MAE	MSE	RMSE	R^2
DNN	11.599±0.578	351.135±12.792	18.34±0.337	0.91±0.007
DNN-DEEPSEEK-R1	13.833±0.232	308.591±12.162	16.608±0.647	0.925±0.003
DNN-GEMMA2	18.724±0.433	676.414±44.572	25.458±0.841	0.845±0.006
DNN-LLAMA3.1	8.661±0.522	190.212±8.852	13.446±0.292	0.953±0.002
DNN-LLAVA	9.729±0.46	208.659±8.362	14.19±0.285	0.943±0.008
DNN-MISTRAL	12.108±0.468	299.242±21.174	17.097±0.589	0.918±0.006
LightGBM	11.49±0.187	421.015±23.279	19.973±0.562	0.903±0.004
LightGBM-DEEPSEEK-R1	21.288±0.412	1179.414±52.86	33.309±0.733	0.738±0.011
LightGBM-GEMMA2	19.157±0.509	958.129±37.932	29.31±1.064	0.783±0.01
LightGBM-LLAMA3.1	20.389±0.426	1095.362±50.1	32.054±0.723	0.75±0.006
LightGBM-LLAVA	19.632±0.539	1105.855±73.842	32.218±1.063	0.75±0.008
LightGBM-MISTRAL	18.277±0.33	1092.098±63.548	31.506±0.875	0.755±0.011
SVM	20.709±0.664	1199.3±47.226	33.881±0.665	0.72±0.008
SVM-DEEPSEEK-R1	16.183±0.503	634.455±83.485	24.195±1.566	0.817±0.026
SVM-GEMMA2	16.022±0.387	658.367±40.724	25.716±0.479	0.854±0.009
SVM-LLAMA3.1	15.658±0.311	692.79±27.78	24.814±0.897	0.836±0.004
SVM-LLAVA	16.827±0.278	806.762±37.487	27.481±0.607	0.813±0.006
SVM-MISTRAL	14.194±0.574	567.195±65.942	23.062±1.329	0.857±0.01
XGBoost	10.719±0.148	322.971±10.993	17.606±0.294	0.926±0.006
XGBoost-DEEPSEEK-R1	20.992±0.489	1005.501±54.987	30.982±0.807	0.777±0.009
XGBoost-GEMMA2	18.73±0.244	803.921±26.879	25.585±1.463	0.81±0.009
XGBoost-LLAMA3.1	19.411±0.294	913.504±38.536	29.555±0.603	0.786±0.005
XGBoost-LLAVA	19.677±0.406	886.024±52.555	29.038±0.845	0.793±0.005
XGBoost-MISTRAL	17.59±0.342	787.674±38.996	27.113±0.636	0.815±0.006

Table S2. Metrics report for validation data of Droplet Diameter (μm) of 1st dataset.

Validation data				
Model	Droplet Generation Rate (Hz)			
	Metrics			
	MAE	MSE	RMSE	R^2
DNN	25.63±0.457	1611.718±103.733	39.486±1.182	0.904±0.011
DNN-DEEPSEEK-R1	12.653±0.283	562.148±80.544	22.206±1.314	0.956±0.009

DNN-GEMMA2	32.331±0.735	3080.966±154.246	53.46±0.992	0.818±0.004
DNN-LLAMA3.1	14.276±0.337	588.468±63.907	23.316±1.515	0.964±0.006
DNN-LLAVA	15.467±0.866	926.915±195.809	28.065±2.639	0.94±0.008
DNN-MISTRAL	17.056±0.73	1132.787±174.44	32.607±2.637	0.942±0.008
LightGBM	19.536±0.6	1426.793±202.113	37.404±1.181	0.908±0.013
LightGBM-DEEPSEEK-R1	33.889±0.987	3853.952±528.915	60.224±3.504	0.765±0.013
LightGBM-GEMMA2	33.296±0.655	3449.437±532.65	59.597±1.607	0.805±0.02
LightGBM-LLAMA3.1	29.857±0.679	2957.485±384.621	52.543±5.724	0.829±0.015
LightGBM-LLAVA	36.222±1.324	4648.347±232.385	65.665±1.749	0.737±0.009
LightGBM-MISTRAL	31.742±0.941	3925.13±323.322	59.876±2.091	0.805±0.022
SVM	40.921±0.701	4930.259±185.426	68.487±1.309	0.669±0.053
SVM-DEEPSEEK-R1	25.493±1.096	3529.95±667.97	57.29±4.978	0.781±0.036
SVM-GEMMA2	29.653±0.863	3945.536±212.754	60.433±1.526	0.782±0.01
SVM-LLAMA3.1	27.548±0.657	3200.001±304.973	53.029±2.543	0.835±0.016
SVM-LLAVA	28.725±0.655	3649.985±787.572	49.042±2.834	0.794±0.032
SVM-MISTRAL	22.977±1.287	2225.636±259.196	44.918±3.021	0.788±0.068
XGBoost	17.49±0.464	1150.849±209.332	30.023±1.604	0.933±0.009
XGBoost-DEEPSEEK-R1	35.394±0.972	3481.008±241.61	56.512±2.267	0.786±0.008
XGBoost-GEMMA2	32.157±0.69	3118.151±128.229	54.302±1.1	0.813±0.009
XGBoost-LLAMA3.1	26.53±0.933	2215.846±199.078	45.065±1.924	0.858±0.008
XGBoost-LLAVA	35.522±0.994	3648.844±264.877	58.425±2.17	0.786±0.014
XGBoost-MISTRAL	31.063±0.582	2813.729±197.27	52.538±1.714	0.804±0.025

Table S3. Metrics report for validation data of Droplet Generation Rate (Hz) of 1st dataset.

Validation data					
Model	Droplet Regime				
	Metrics				
	Accuracy	F1 Score	Precision	Recall	ROC AUC
DNN	0.96±0.003	0.951±0.008	0.957±0.006	0.948±0.008	0.958±0.006
DNN-DEEPSEEK-R1	0.968±0.003	0.962±0.003	0.956±0.008	0.959±0.009	0.968±0.003
DNN-GEMMA2	0.956±0.006	0.947±0.006	0.965±0.004	0.96±0.005	0.955±0.006
DNN-LLAMA3.1	0.977±0.006	0.972±0.003	0.969±0.011	0.965±0.005	0.976±0.003
DNN-LLAVA	0.96±0.006	0.954±0.003	0.955±0.005	0.952±0.004	0.961±0.003
DNN-MISTRAL	0.968±0.002	0.96±0.003	0.97±0.01	0.956±0.006	0.969±0.002
LightGBM	0.962±0.01	0.954±0.011	0.975±0.008	0.953±0.007	0.959±0.01
LightGBM-DEEPSEEK-R1	0.948±0.005	0.938±0.008	0.949±0.006	0.938±0.008	0.933±0.01
LightGBM-GEMMA2	0.951±0.002	0.941±0.006	0.954±0.004	0.919±0.013	0.949±0.003
LightGBM-LLAMA3.1	0.961±0.006	0.953±0.008	0.957±0.005	0.936±0.005	0.958±0.007

LightGBM-LLAVA	0.948±0.004	0.937±0.004	0.957±0.009	0.917±0.005	0.944±0.003
LightGBM-MISTRAL	0.943±0.004	0.935±0.006	0.957±0.004	0.909±0.008	0.939±0.004
SVM	0.958±0.01	0.957±0.003	0.97±0.004	0.947±0.004	0.956±0.01
SVM-DEEPSEEK-R1	0.957±0.002	0.948±0.003	0.952±0.008	0.95±0.005	0.956±0.003
SVM-GEMMA2	0.956±0.006	0.949±0.006	0.946±0.005	0.954±0.01	0.956±0.006
SVM-LLAMA3.1	0.96±0.006	0.94±0.012	0.957±0.005	0.941±0.012	0.95±0.01
SVM-LLAVA	0.944±0.012	0.944±0.003	0.939±0.018	0.952±0.004	0.948±0.007
SVM-MISTRAL	0.958±0.007	0.951±0.008	0.95±0.012	0.954±0.016	0.958±0.007
XGBoost	0.964±0.006	0.956±0.007	0.975±0.006	0.956±0.006	0.963±0.007
XGBoost-DEEPSEEK-R1	0.94±0.008	0.942±0.006	0.945±0.009	0.941±0.011	0.942±0.003
XGBoost-GEMMA2	0.947±0.003	0.936±0.005	0.953±0.004	0.917±0.011	0.942±0.005
XGBoost-LLAMA3.1	0.955±0.006	0.946±0.007	0.952±0.006	0.935±0.005	0.947±0.01
XGBoost-LLAVA	0.95±0.003	0.938±0.005	0.955±0.014	0.905±0.007	0.944±0.003
XGBoost-MISTRAL	0.942±0.003	0.937±0.005	0.956±0.004	0.909±0.006	0.938±0.003

Table S4. Metrics report for validation data of Droplet Regime of 1st dataset.

Validation data				
Model	Droplet Diameter (μm)			
	Metrics			
	MAE±SE	MSE±SE	RMSE±SE	R^2±SE
DNN	8.145±0.153	148.119±5.079	11.94±0.182	0.914±0.004
DNN-DEEPSEEK-R1	4.604±0.152	55.958±3.227	7.296±0.213	0.968±0.002
DNN-GEMMA2	6.645±0.131	123.051±5.007	10.788±0.211	0.933±0.003
DNN-LLAMA3.1	6.03±0.101	90.436±3.809	9.259±0.254	0.95±0.002
DNN-LLAVA	5.729±0.14	96.793±7.24	9.668±0.279	0.952±0.002
DNN-MISTRAL	4.988±0.089	72.74±3.76	8.211±0.188	0.961±0.003
LightGBM	4.885±0.152	86.67±7.428	9.092±0.366	0.958±0.003
LightGBM-DEEPSEEK-R1	9.642±0.194	324.416±18.161	19.286±1.27	0.832±0.006
LightGBM-GEMMA2	11.349±0.356	404.349±29.26	18.298±0.408	0.816±0.005
LightGBM-LLAMA3.1	10.865±0.264	450.262±21.228	20.391±0.496	0.769±0.016
LightGBM-LLAVA	10.562±0.262	500.824±61.228	20.588±0.89	0.788±0.014
LightGBM-MISTRAL	11.467±0.578	426.522±20.15	22.114±1.46	0.771±0.027
SVM	6.916±0.137	143.021±5.682	11.772±0.275	0.918±0.004
SVM-DEEPSEEK-R1	7.273±0.148	162.185±14.972	12.593±0.6	0.918±0.006
SVM-GEMMA2	8.601±0.314	224.91±27.778	14.571±0.793	0.875±0.03
SVM-LLAMA3.1	10.11±0.348	267.952±24.473	15.549±0.514	0.877±0.009
SVM-LLAVA	10.12±0.223	224.957±8.644	14.734±0.281	0.872±0.005
SVM-MISTRAL	9.38±0.656	273.189±44.016	15.977±1.338	0.865±0.01

XGBoost	4.799±0.274	71.16±3.828	8.143±0.21	0.962±0.002
XGBoost-DEEPSEEK-R1	9.907±0.43	289.776±42.554	16.549±1.262	0.864±0.005
XGBoost-GEMMA2	10.195±0.265	336.621±32.529	17.48±1.386	0.849±0.009
XGBoost-LLAMA3.1	11.692±0.593	421.171±51.241	18.29±0.403	0.82±0.006
XGBoost-LLAVA	10.6±0.46	359.326±62.12	18.32±1.539	0.837±0.012
XGBoost-MISTRAL	10.316±0.355	341.157±28.096	16.995±0.393	0.841±0.004

Table S5. Metrics report for validation data of Droplet Diameter (µm) of 2nd dataset.

Validation data				
Model	Droplet Generation Rate (Hz)			
	Metrics			
	MAE	MSE	RMSE	R^2
DNN	648.523±18.432	1244960.634±46369.189	1091.635±23.125	0.874±0.006
DNN-DEEPSEEK-R1	330.433±5.544	294417.841±9841.107	538.615±18.32	0.966±0.001
DNN-GEMMA2	451.315±9.441	519943.456±19758.248	708.275±12.894	0.941±0.002
DNN-LLAMA3.1	309.975±5.948	343880.144±20537.965	562.228±14.286	0.96±0.002
DNN-LLAVA	347.215±9.928	442701.833±31933.129	641.895±23.178	0.948±0.004
DNN-MISTRAL	326.891±6.419	353665.142±16019.408	578.045±12.757	0.959±0.002
LightGBM	341.659±7.632	546316.728±83943.298	712.434±62.253	0.934±0.01
LightGBM-DEEPSEEK-R1	518.394±22.854	853308.939±34266.26	896.477±18.191	0.904±0.003
LightGBM-GEMMA2	600.551±19.269	1344118.755±59665.758	1104.896±24.27	0.848±0.007
LightGBM-LLAMA3.1	609.056±14.062	1346304.657±63780.94	1181.12±104.16	0.833±0.012
LightGBM-LLAVA	548.174±14.053	1234899.968±63686.555	1039.502±50.208	0.866±0.008
LightGBM-MISTRAL	520.621±16.224	1067934.765±146682.105	1059.27±46.136	0.869±0.009
SVM	634.444±21.018	1187059.703±205305.937	1100.073±27.854	0.86±0.005
SVM-DEEPSEEK-R1	483.062±10.98	851717.649±41268.379	946.633±63.225	0.892±0.011

SVM-GEMMA2	544.937±13.619	1028167.92±169237.571	1015.691±60.26	0.876±0.009
SVM-LLAMA3.1	562.845±18.857	1110619.828±54766.613	1014.106±24.313	0.876±0.005
SVM-LLAVA	584.966±14.053	1091501.206±53557.942	994.406±88.343	0.878±0.004
SVM-MISTRAL	519.165±11.946	968253.517±169217.175	968.309±27.479	0.887±0.016
XGBoost	296.543±22.406	409808.935±64018.947	629.418±18.694	0.951±0.002
XGBoost-DEEPSEEK-R1	494.26±9.014	992700.746±112539.143	917.949±24.206	0.9±0.004
XGBoost-GEMMA2	588.324±11.125	1114132.834±188188.467	1011.69±21.49	0.869±0.009
XGBoost-LLAMA3.1	606.936±13.367	1152314.469±63458.753	1029.97±19.266	0.866±0.008
XGBoost-LLAVA	534.05±10.327	996351.982±134347.393	975.792±66.469	0.892±0.004
XGBoost-MISTRAL	522.069±18.601	965118.308±142584.272	955.915±24.711	0.893±0.005

Table S6. Metrics report for validation data of Droplet Generation Rate (Hz) of 2nd dataset.

Validation data				
Model	Droplet Capillary Number			
	Metrics			
	MAE	MSE	RMSE	R^2
DNN	0.098±0.004	0.024±0.001	0.153±0.002	0.771±0.008
DNN-DEEPSEEK-R1	0.146±0.007	0.04±0.004	0.203±0.007	0.612±0.042
DNN-GEMMA2	0.084±0.002	0.016±0.001	0.138±0.002	0.827±0.012
DNN-LLAMA3.1	0.128±0.002	0.032±0.001	0.175±0.002	0.699±0.016
DNN-LLAVA	0.088±0.002	0.023±0.002	0.15±0.002	0.786±0.015
DNN-MISTRAL	0.119±0.002	0.029±0.001	0.167±0.002	0.745±0.023
LightGBM	0.08±0.002	0.021±0.002	0.152±0.005	0.777±0.018
LightGBM-DEEPSEEK-R1	0.109±0.002	0.031±0.001	0.173±0.002	0.708±0.006
LightGBM-GEMMA2	0.098±0.002	0.028±0.001	0.167±0.003	0.728±0.007
LightGBM-LLAMA3.1	0.112±0.003	0.032±0.002	0.17±0.009	0.72±0.017
LightGBM-LLAVA	0.11±0.002	0.031±0.002	0.177±0.002	0.694±0.006

LightGBM-MISTRAL	0.101±0.003	0.029±0.001	0.169±0.003	0.723±0.009
SVM	0.093±0.002	0.022±0.001	0.146±0.002	0.803±0.02
SVM-DEEPSEEK-R1	0.115±0.001	0.029±0.001	0.17±0.002	0.744±0.015
SVM-GEMMA2	0.114±0.004	0.026±0.002	0.162±0.004	0.752±0.015
SVM-LLAMA3.1	0.12±0.002	0.03±0.002	0.169±0.005	0.705±0.01
SVM-LLAVA	0.119±0.003	0.035±0.001	0.184±0.003	0.684±0.016
SVM-MISTRAL	0.105±0.003	0.026±0.002	0.162±0.004	0.734±0.017
XGBoost	0.09±0.002	0.022±0.001	0.148±0.002	0.786±0.014
XGBoost-DEEPSEEK-R1	0.123±0.003	0.034±0.001	0.184±0.003	0.676±0.011
XGBoost-GEMMA2	0.106±0.003	0.029±0.002	0.17±0.006	0.726±0.009
XGBoost-LLAMA3.1	0.117±0.003	0.03±0.002	0.172±0.005	0.712±0.034
XGBoost-LLAVA	0.116±0.003	0.03±0.002	0.173±0.006	0.709±0.02
XGBoost-MISTRAL	0.106±0.003	0.029±0.002	0.167±0.005	0.723±0.007

Table S7. Metrics report for validation data of Droplet Capillary Number of 1st dataset.
